# Who qualifies to be a bioinformatician?

**DOI:** 10.3389/fgene.2015.00164

**Published:** 2015-04-24

**Authors:** Antony T. Vincent, Steve J. Charette

**Affiliations:** ^1^Institut de Biologie Intégrative et des Systèmes, Université LavalQuebec City, QC, Canada; ^2^Département de Biochimie, de Microbiologie et de Bio-Informatique, Faculté des Sciences et de génie, Université LavalQuebec, QC, Canada; ^3^Centre de Recherche de l'Institut Universitaire de Cardiologie et de Pneumologie de QuébecQuebec City, QC, Canada

**Keywords:** bioinformatician, bioinformatics, biologist, informatician, scientist

## The bioinformatics

Like microscopes and thermal cyclers, computers are routinely used in many laboratories. Bioinformatics is a recent scientific discipline that has undergone strong and rapid progression and evolution (Ouzounis, 2012). The use of bioinformatics analyses in biological studies in fields as diverse as metagenomics (Hurwitz et al., 2014) and infectious diseases (Gire et al., 2014) is now accepted and viewed as normal.

As mentioned in *PLoS computational biology* by Hogeweg (2011), the first time the term “bioinformatics” was used was in 1970 in a Dutch article. At the time, bioinformatics referred to “the study of informatic processes in biotic systems.” Since then, bioinformatics has gradually carved out a place for itself in the scientific community with, for example, the creation in 1985 of *CABIOS* (Computer Applications in the Biosciences), which is now known as *Bioinformatics* (Oxford, England). However, the main impulse for the emergence of bioinformatics came from the completion of the human genome project at beginning of the new century. However, it also gave rise to a fundamental question. What exactly is bioinformatics? Because of the importance of bioinformatics, this neologism was quickly added to the Oxford English Dictionary (OED), and discussions about the definition of bioinformatics also heated up (Luscombe et al., 2001). According to the OED, bioinformatics is “the branch of science concerned with information and information flow in biological systems, especially the use of computational methods in genetics and genomics.” While this definition is very broad and can be unclear and somewhat open to interpretation, the definition of bioinformatician is even less clear: “An expert in or practitioner of bioinformatics.” Because bioinformatics is carving out an increasingly important place in research and because we have to help students to understand their future role in research, a simple but complex question came to mind: Who qualifies to be a bioinformatician?

To attempt to answer this question, let us start with a simple observation. In the past few years, there has been an explosion in bioinformatics tools, some are free and are under a public license (Vincent and Charette, 2014) while others are proprietary and are sometimes distributed by companies (Smith, 2014). In the early years of bioinformatics, the tools were mainly command lines and were less accessible to neophytes. The people developing and using these tools were mainly considered bioinformaticians, that is, people with sufficient skills in informatics and biology to use the tools and analyze the results. However, in fact, bioinformaticians designed these tools for themselves not for biologists, which caused a certain degree of discontent in the scientific community (Kumar and Dudley, 2007). However, powerful and much simpler tools are now available with an easy-to-understand interface, including NCBI Blast (Johnson et al., 2008), Unipro UGENE (Okonechnikov et al., 2012), the web server CONTIGuator (Galardini et al., 2011), the genome viewer Artemis (Rutherford et al., 2000), and many others.

These tools have provided biologists with user-friendly bioinformatics tools. Since many biologists now conduct sophisticated bioinformatics analyses, can they be called bioinformaticians? This is not an easy question to answer, in part because of the broad definition of bioinformatics.

## The two conceptual aspects of bioinformatics

At the very beginning of his book *Perl Programming for Biologists* (Jamison, 2003), Curtis D. Jamison differentiates between two conceptual aspects of bioinformatics: computational biology and analytical bioinformatics. Computational biology uses algorithms to mathematically (statistically) analyze biological problems and tries to build a model to infer solutions using a computational approach. On the other hand, analytical bioinformatics uses bioinformatics tools to conduct analyses in a biological context. Consequently, we can reformulate the question posed above in a more accurate way. Can people working in the fields of computational biology or analytical bioinformatics be considered bioinformaticians?

## The bioinformatician

We are aware that gray zones exist and will likely always exist, even as the field of bioinformatics evolves. However, it will be easier to provide an answer to our question. As for the definition provided by OED, we propose that bioinformaticians are experts in the field of bioinformatics. They may be users, but this is not enough to consider them as bioinformaticians (i.e., an expert). Bioinformaticians are scientists who develop and conduct research based on a bioinformatics approach, they do not just use the tools to better understand a biological problem. It is a little like saying that driving your car to work does not make you a mechanic. A bioinformatician is a scientist who understands the underlying “mechanics” of bioinformatics or, more realistically, an aspect of bioinformatics (genomics, protein structure predictions, phylogenetic models, etc.). In a more conceptual framework, bioinformaticians can perhaps be seen as the “missing link” required for improving multidisciplinary research. Since they can bridge biological sciences, informatics, and mathematics, fully fledged bioinformaticians can be valuable assets for multidisciplinary studies. For example, more and more bioinformaticians are becoming involved in major multidisciplinary studies such as those on cancer (Hanauer et al., 2007; Valencia and Hidalgo, 2012) as well as in whole-exome sequencing (WES), which is an increasingly important method used in medical studies (Sanders et al., 2012; Wang et al., 2013; Zhu et al., 2015).

In fact, we are probably able to separate the bioinformaticians in two categories which are not mutually exclusives: (1) the developers who are working directly on algorithms (conception), the development aspects and the maintenance of tools and (2) the curators who architecturally design and maintain data resources and provide an integration of the curated data. There are great bioinformaticians for example at NCBI (http://www.ncbi.nlm.nih.gov), EMBL (http://www.ebi.ac.uk), and *The Comprehensive Antibiotic Resistance Database* (CARD) (McArthur et al., 2013), who maintain and curate databases and others who are developing and maintaining the different tools. These databases and others need bioinformaticians who are skilled in both informatics and biology and who can provide a link between the various tools and the data and who can validate the entries in order to maintain a high level of scientific rigor.

Consequently, in our opinion a biologist who only uses bioinformatics tools to perform analyses but does not contribute at the conception of such tools or not fits in the curator definition provided above is not a bioinformatician. She or he may use the tools proficiently, but as a user not as a bioinformatician. In fact, a strict user of bioinformatics tools could be an expert in another field, for example a genomicist can uses bioinformatics tools, without being a bioinformatician. But, what about the flip side of the coin: a bioinformatician who focuses on informatics problems? We believe that it is easier for a bioinformatician to become an informatician. However, the term bioinformatics encompasses two concepts: “bio,” which refers to biological sciences, and “informatics,” which refers to computational sciences. Just like a biologist is not a bioinformatician, an informatician is not a bioinformatician. It is important to keep in mind that bioinformatics has to be applied in a biological context. For example, maintaining a biological web server (without a curating aspect) is not a bioinformatics task. Informaticians with networking and programming language (SQL, HTML, Python) skills can do the job. It could be a part of a bioinformatician's job, but it should not be the only part of his or her job, otherwise the bioinformatician becomes an informatician.

## The importance of a clearer definition

As bioinformatics gains in importance, it is crucial that the concept of bioinformatician be clearly defined. A clear definition will help universities to adapt their bioinformatics programs to their true needs and to produce real bioinformaticians with the proper skills. This will also help human resources departments to improve the accuracy of job descriptions and avoid the many knotty administrative issues involved in defining tasks, categorizing employees for union purposes and perhaps, most importantly, recognizing and certifying bioinformaticians. Like virus taxonomy, a good definition of a bioinformatician should not be based on a single concept but should be polythetic, this is, real bioinformaticians share a number of common characteristics, but none of which is essential.

Many university departments, including ours, now give mandatory bioinformatics courses to students enrolled in biology, biochemistry, and microbiology programs, among others. This is essential in a context where these students will be called on to use bioinformatics tools and the results provided by them during their careers. However, it is also important for students to realize that a 45-h bioinformatics course will not make them experts in the field or qualify them as bioinformaticians. Much more training will be needed to reach that goal.

The goal of this paper is thus to contribute to the discussion of how best to define people working in a constantly evolving field like bioinformatics, which in turn is part of the larger discipline of computational science. As for bioinformatics, other sciences such as physics, mathematics, and chemistry will probably also have to evolve and adapt at this emerging and important field.

### Conflict of interest statement

The authors declare that the research was conducted in the absence of any commercial or financial relationships that could be construed as a potential conflict of interest.
